# Comparison of R5 and G12 Antibody-Based ELISA Used for the Determination of the Gluten Content in Official Food Samples

**DOI:** 10.3390/foods4040654

**Published:** 2015-11-09

**Authors:** Rupert Hochegger, Walter Mayer, Manuela Prochaska

**Affiliations:** Austrian Agency for Health and Food Safety (AGES), Institute for Food Safety, Vienna 1220, Austria; E-Mails: walter.mayer@ages.at (W.M.); manuela.prochaska@ages.at (M.P.)

**Keywords:** ELISA, gluten analysis, G12 antibody, R5 antibody, gluten-free, gluten sensitivity, celiac disease

## Abstract

Celiac Disease (CD) is one of the most common food intolerances. It comes along with serious damage of the mucosa in the small intestine and is caused by the storage proteins—termed “gluten”—of wheat, rye, barley and possibly oats. Sensitive individuals need to stick to a strict gluten-free diet. The gluten level in food products labeled as “gluten-free”, must not exceed 20 mg/kg. It is obvious that effective test methods are needed to accurately determine the gluten concentration in foods. The determination of the presence of gluten in foodstuffs is mainly done by means of an immunochemical method called ELISA (enzyme-linked immunosorbent assay). To check the suitability of a G12 antibody-based gluten detection kit for its use in official control systems a number of routine samples were tested in parallel with two different test kits, as would be done in a routine lab. The determination of the gluten content was performed on samples entering the official laboratory including samples from official control plans, commercially available and private samples to request gluten-free labels. The results obtained with the G12 antibody ELISA assay were comparable to the official R5 method. A validation of the two different methods was not part of this study.

## 1. Introduction

Although grains normally are part of a balanced diet, an increasing number of people are becoming more sensitive to them, developing clinical conditions like wheat allergies, gluten sensitivity, wheat-dependent exercise induced anaphylaxis (WDEIA), and celiac disease.

Gluten—the main group of proteins in grains such as wheat, rye, barley, and to a lesser extent oats—play a crucial role in the development of celiac disease. This auto-immune disorder of genetically predisposed individuals, affects about 1% of the world’s population and is caused by the ingestion of gluten proteins [1]. The resistance of some gluten proteins, especially prolamins, to proteases and peptidases in the stomach and gut, and further deamidation of those proteins leads to the development of T-cell epitopes, stimulating the immune system. The consequence is inflammation and destruction of villi in the small intestine, which in the long-term leads to malnutrition. Sensitive individuals need to stick to a strict gluten-free diet due to the fact that there is no cure to celiac disease at the moment [2].

Compliance with this gluten-free diet can often be challenging for the affected persons. In addition to the learning process involved in determining which products should be avoided, celiac patients are highly reliant on the correct labeling of products. The fact that gluten is used extensively in the food industry due to its functional properties, adds further complexity to this issue. Without an adequate allergen management plan incorporated to the HACCP (hazard analysis critical control points), the unintentional contamination of products during the production process can lead to a misbranding of products and thus threaten any gluten-sensitive people. According to Codex Alimentarius (CODEX STAN 118-1979) [3], European Commission Regulation (EC 41/2009) [4] and the U.S. Food and Drug Administration (FR Doc. 2013-18813) [5], products labeled as “gluten-free” are not allowed to contain more than 20 mg/kg of gluten.

Immunoassays like ELISAs (enzyme-linked immunosorbent assays) and LFDs (lateral flow devices) have many advantages, and are therefore frequently used in the food industry to track allergen contamination. However, one big issue in allergen quantification is that results vary from kit to kit. The lack of an official reference method and official reference materials are factors contributing to this issue. Various technical differences such as antibody specificity, target analytes, sample extraction buffers, extraction time and temperature, calibration standards, and the unit of measurement are the other main factors. Furthermore, different matrixes and their different processing statuses add to the complexity of allergen testing. In particular, thermal processing during production can lead to altered protein extraction efficiencies and antibody binding affinities [6].

The current Type I Codex method for gluten analysis is the ELISA R5 Mendez method, which is calibrated against the Prolamin Working Group (PWG) Gliadin standard and used by official control systems throughout Europe [7]. The aim of this study was to compare the suitability of an ELISA G12 method on routine samples entering the official laboratory of AGES (Agentur für Gesundheit und Ernährungssicherheit GmbH)—part of the official food control system in Austria. The different antibodies (R5: raised against omega-Secalin from rye/G12: raised against alpha 2-gliadin 33 mer from wheat) used in these assays are not the only differences. The ELISA G12 method is calibrated with vital wheat gluten (VWG) solution from Roquette, Corby, Northants, UK. Furthermore, the kits differ in their limits of detection (LOD, 2 mg/kg gluten for the G12 assay and 3 mg/kg gluten for the R5 assay), as well as in their quantification ranges (4–200 mg/kg gluten for the G12 assay and 5–80 mg/kg gluten for the R5 assay).

## 2. Experimental Section

### 2.1. Samples

Routine samples from different types of food, all labelled as gluten-free but with a gluten content above LOQ (LOQ = 2.5 mg/kg Gliadin, R5 ELISA) were collected from Austrian supermarkets, retailers, and producers, or received from manufacturers. Samples were extracted and analyzed according to the standard procedure described below. All samples were analyzed in duplicates.

### 2.2. Sample Extraction Procedure

For the extraction of samples, no commercial kit procedure was used. Instead, an in-house modified extraction method was applied that combines the ethanol and cocktail extraction, described below:

To overcome problems frequently observed when testing real life samples, 5 g of homogenized material were used for the extraction procedure to reduce the impact of possible heterogeneity in our samples. Therefore, an ethanol extraction step was carried out prior to the treatment with the cocktail solution as suggested by the kit provider. After homogenization 25 mL of ethanol 60% is added to 5 g of each sample. After 3 h of incubation at 40 °C (Unihood 750, Uniequip, Martinsried, Germany) the samples are centrifuged at 460 *g* for 10 min (model 5810 R, Eppendorf, Hamburg, Germany) and the supernatants stored in screw top vials. The precipitates are further diluted by 6.25 mL of the cocktail solution and incubated for 40 min at 50 °C. Finally, 18.75 mL of ethanol 80% are added to the sample and incubated at room temperature for an hour while shaking every 10–15 min. After centrifugation at 3220 *g* for 10 min both supernatants are pooled. The cocktail was prepared according to the recipe of the patented Mendez Cocktail Solution (EP 2003448 A1), Dithiothreitol (DTT) was used as reducing agent.

### 2.3. Test Design

The aim of this study was to evaluate the suitability of a G12 antibody based gluten detection kit for the use in an official control system, by testing a number of routine samples. Therefore, samples entering the official laboratory were assayed with two different test kits in parallel—the G12 antibody test kit (Romer Labs) and the current R5 Codex Alimentarius type 1 method (R-Biopharm), which is accredited and has run for more than 10 years at AGES. Since the extraction of proteins from samples is the crucial step in this kind of analysis, we decided to use an identical extraction procedure (described above) prior to assay runs to eliminate side errors. Protein extracts were used immediately after extraction followed by the two individual assay procedures (a comparison of the two different assay procedures can be found in Table 1). Each assay run was accompanied by proven material (*i.e.*, proficiency test material, the extraction step was included here) and a positive (PWG Gliadin standard) and a negative control (blank). The measurement uncertainty was determined with 30%.

**Table 1:** Comparison of the two different assay procedures.

	R5 Method	G12 Method
Addition of standard/samples to wells	100 µL of each standard solution or sample	Add 100 µL of each standard solution or sample
1st incubation	30 min at room temperature (RT)	20 min at room temperature (RT)
Washing	Empty the contents of the microwell strips	Empty the contents of the microwell strips
	Wash by filling each microwell with 250 μL diluted wash buffer, and then emptying the buffer from the microwell strips.	Wash by filling each microwell with 300 µL diluted wash buffer, and then emptying the buffer from the microwell strips.
	Repeat this step a total of 3 times.	Repeat this step a total of 5 times.
Addition of conjugate and 2nd incubation	Add 100 μL of the diluted enzyme conjugate to each well and incubate for 30 min at room temperature (20–25 °C/68–77 °F).	Add 100 μL of the diluted enzyme conjugate to each well and incubate for 20 min at room temperature.
Washing	Repeat washing step as described above	Repeat washing step as described above
Addition of substrate and 3rd incubation	Add 50 μL of substrate and 50 μL of chromogen to each well. Mix gently by shaking the plate manually and incubate for 30 min at room temperature (20–25 °C/68–77 °F) in the dark.	Pipette 100 µL of the Substrate into each microwell and incubate at room temperature for 20 min in the dark.
Additions of stop solution and measurement	Add 100 μL of the stop reagent to each well. Mix gently by shaking the plate manually and measure the absorbance at 450 nm. Read within 30 min after addition of stop solution.	Pipette 100 µL of Stop Solution into each microwell. The color should change from blue to yellow. Read the strips with a microwell reader using a 450 nm filter. Record OD readings for each microwell.

## 3. Results and Discussion

### 3.1. Statistical Analysis

Statistical analyses were performed with Microsoft Excel (version 14.0.7140.5002) and QuickCalcs (www.graphpad.com/quickcalcs).

For evaluation samples were clustered into four samples matrix groups, namely: Flour, bakery products, soy products and processed food. Each product group was analyzed separately using the dependent *t*-test for paired samples. Statistical significance was defined by a 1-sided alpha value of 0.05. Sample pairs where one or both test kits gave results <LOQ were excluded from the *t*-test because no true value could be assigned. Finally all results (even the ones giving results <LOQ) were rated in regards to the official 20 mg/kg threshold for gluten-free products, with results below 20 mg/kg being negative (N) and results with 20 mg/kg or more being positive (P). The resulting rated pairs (N-N, P-P, N-P, P-N) were analyzed using the McNemar’s test from QuickCalcs. Statistical significance was defined by a 2-sided alpha value of 0.05.

FAPAS (food analysis performance assessment scheme) proficiency test material was carried along with each assay, but not included in the statistical analysis of the product groups. The results of these samples are depicted in Table 2 below.

**Table 2:** Results of the food analysis performance assessment scheme (FAPAS) proficiency test material.

FAPAS Control Sample	R5 ELISA Gluten (mg/kg)	G12 ELISA Gluten (mg/kg)
FAPAS 2781B—flour group test run	33.1	32.1
FAPAS 2781B—bakery group test run	26.1	26.0
FAPAS 2781B—soy/processed food group test run (gluten 1:2)	13.5	13.8

#### 3.1.1. Flour Group

**Figure 1:**
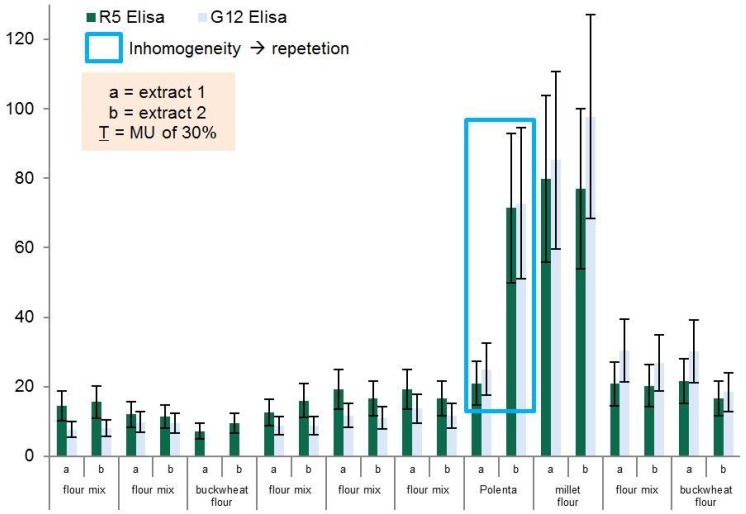
Results for the product group flour.

The flour group consists of 10 samples which are depicted in Table 3 and Figure 1. The buckwheat flour sample which gave results below LOQ with both methods was excluded from *t*-test analysis as no real values could be assigned.

**Table 3:** Results of the flour product group: The buckwheat flour sample (highlighted in bold), which produced results below the LOQ for both methods, was excluded from the statistical analysis (*n* = number of samples, df = degrees of freedom).

Product Group—Flour		R5 ELISA Gluten (mg/kg)	G12 ELISA Gluten (mg/kg)
Flour mix		15.0	7.8
Flour mix		11.6	9.6
**Buckwheat flour**		**<5**	**<4**
Flour mix		14.4	8.7
Flour mix		17.9	11.3
Flour mix		17.9	12.7
Polenta		46.2	48.9
Millet flour		78.4	91.4
Flour mix		20.8	28.6
Buckwheat flour		19.1	24.3
**Statistics**	Mean	26.8	27.0
	*n*	9	9
	df	8	
	***p*-value one-sided**	**0.4623**	
	*p*-value two-sided	0.9246	

Most of the samples in the flour group were in the range of 20 mg/kg of gluten. Only the polenta and the millet flour sample had gluten values well above 20 mg/kg. For the polenta, an average value was assumed as the test results were the same after repeated measurements.

Figure 1 demonstrates the robustness of both methods nicely, as there is only a small deviation when comparing the results of extract a and b within one matrix with the same method. The only large deviation between extract a and b observed is for the polenta sample. But as this variation is observed both for the R5 and for the G12 method. This inhomogeneity was probably caused by a mistake during extraction and, therefore, this sample’s test needs to be repeated. The high test results in the millet flour sample in both methods for both extracts a and b can only be explained by a contamination and thus, the mislabeling of the respective product. But aside from these two samples, the flour product group showed homogenous results within the range of 30% measurement uncertainty (error bars) and no significant differences between the two test kits could be observed in this product group (*p*-value one-sided = 0.4623; *n* = 9).

#### 3.1.2. Bakery Products Group

**Figure 2:**
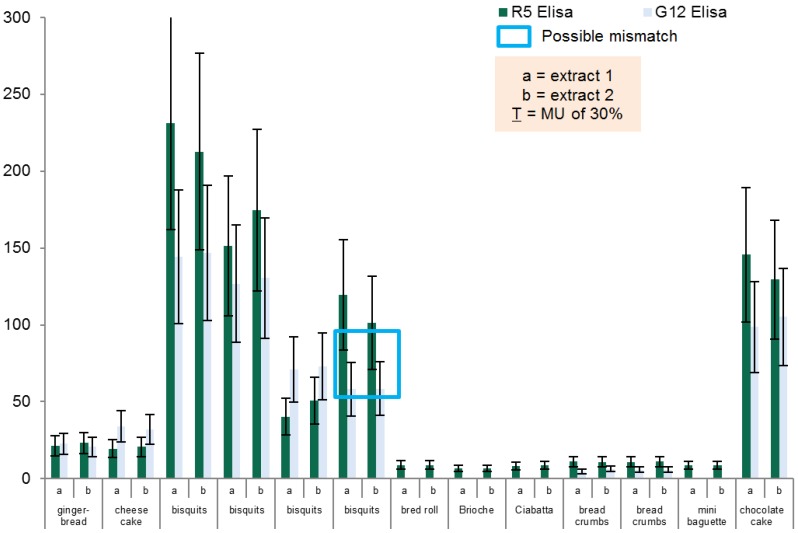
Results for the group of bakery products.

The bakery product group was the biggest group, consisting of 19 samples which are depicted in Table 4 and Figure 2. Nine Samples had to be excluded from the *t*-test analysis as one of the test kits gave a result below the LOQ and could, therefore, be not assessed.

The group of bakery products also ranged from around the threshold of 20 mg/kg gluten, with the exception of three biscuit samples and the chocolate cake sample, which showed gluten values well above 20 mg/kg.

Furthermore, it also demonstrates the robustness of both methods nicely, as there is only a small deviation when comparing the results of extract a and b within one matrix with the same method. In the four products showing elevated levels for gluten, only one biscuit sample showed an inhomogeneity when comparing the two methods (see Figure 2—blue box). A significant difference was observed for this biscuit sample with a value of 110.4 mg/kg gluten determined by the R5 ELISA method and a value of 58.4 mg/kg gluten determined with the G12 ELISA.

**Table 4:** Results of the bakery products group. Nine samples (highlighted in bold), which gave results below the LOQ for one or both methods, were excluded from the statistical analysis (*n* = number of samples, df = degrees of freedom).

Product Group—Bakery Products		R5 ELISA Gluten (mg/kg)	G12 ELISA Gluten (mg/kg)
Gingerbread		22.3	21.7
Cheese cake		20.1	33.0
Biscuits		222.1	145.5
Biscuits		162.9	128.6
Biscuits		45.4	71.9
Biscuits		110.4	58.4
**Bred roll**		**9.0**	**<4**
**Brioche**		**6.7**	**<4**
**Ciabatta**		**8.4**	**<4**
Bread crumbs		11.0	5.5
Bread crumbs		11.0	5.9
**Mini baguette**		**8.6**	**<4**
Chocolate cake		137.5	101.9
**Cottage loaf**		**7.7**	**<4**
**Crispbread**		**8.0**	**<4**
Crispbread		10.0	9.9
**Toast**		**8.8**	**<4**
**Cottage loaf**		**7.6**	**<4**
**Brioche**		**6.8**	**<4**
**Statistics**	Mean	75.3	58.2
	*n*	10	10
	df	9	
	***p*-value one-sided**	**0.0615**	
	*p*-value two-sided	0.1230	

The other three samples with elevated gluten levels did not show any significant differences between the two methods in the 30% range of uncertainty. Since both methods, the G12 and R5 ELISA, coincidentally determined high gluten values for those four samples, we suggest that those four samples were initially contaminated in the course of preparation.

Although there was a significant difference in the result of one sample for these methods, no significant difference could be observed when analyzing the whole product group (*p*-value one-sided = 0.0615; *n* = 10).

#### 3.1.3. Soy Products Group

The soy product group consists of seven samples, which are depicted in Table 5. Five out of seven samples had to be excluded from the *t*-test analysis as one or both test kits gave a result below the LOQ, leaving only two samples for analysis, which were too few to carry out a *t*-test.

**Table 5:** Results of the soy products group. Five samples (highlighted in bold) gave results below the LOQ for one or both methods and were excluded from the statistical analysis and, therefore, *t*-test analysis had to be omitted for this product group with only two evaluable results.

Product Group—Soy Products	R5 ELISA Gluten (mg/kg)	G12 ELISA Gluten (mg/kg)
Soy granulate	95.0	95.6
**Soy flour**	**8.4**	**<4**
**Semolina**	**<5**	**<4**
**Semolina**	**7.6**	**<4**
**Semolina**	**6.2**	**<4**
Soy granulate	13.1	4.7
**Semolina**	**<5**	**<4**

For the product group soy products, no significant differences between the two methods could be observed. Only soy granulate showed elevated gluten levels well above 20 mg/kg gluten. Since both methods confirmed this high value, it must be assumed that this sample was contaminated with a gluten source.

#### 3.1.4. Processed Food Group

The processed food group consists of seven samples, which are depicted in Table 6. Two samples had to be excluded from the *t*-test analysis, as one or both test kits gave a result below the LOQ and could, therefore, be not assessed.

**Table 6:** Results of the processed food group. Two samples (highlighted in bold) gave results below the LOQ for one or both methods and were excluded from the statistical analysis (*n* = number of samples, df = degrees of freedom).

Product Group—Processed Food		R5 ELISA Gluten (mg/kg)	G12 ELISA Gluten (mg/kg)
Pizzasnack Margarita		21.5	37.5
**Salt sticks**		**7.8**	**<4**
Spice blend (meat)		25.0	23.7
Spice blend (meat)		22.4	21.5
**Pepper Snack**		**<5**	**<4**
Falafel Mix		12.5	13.9
Spicy sauce		641.9	568.3
**Statistics**	Mean	146.5	132.9
	*n*	5	5
	df	4	
	***p*-value one-sided**	**0.2164**	
	*p*-value two-sided	0.4328	

For the product group processed foods, no significant differences between the two methods could be observed (*p*-value one-sided = 0.2164; *n* = 5). Only spicy sauce showed elevated gluten levels well above 20 mg/kg of gluten. Since both methods confirmed this high value, it must be assumed that this sample was contaminated with a gluten source.

Finally, all the sample results (43 sample pairs) were rated positive or negative, according to the official 20 mg/kg threshold and compared using the McNemar’s test. With this analysis, we were able to include all the results—even the results giving values below the LOQ of a test kit. Results of the McNemar’s test are depicted below in Table 7.

**Table 7:** Results of the McNemar’s test conducted on all the sample pairs.

		G12 ELISA		
		Positive	Negative	Total
**R5 ELISA**	Positive	15	1	**16**
	Negative	1	26	**27**
	**Total**	**16**	**27**	**43**

With a two-tailed *p*-value of 0.4795, no significant difference between the two test kits could be shown. Only two out of 43 samples showed a little discrepancy in their results regarding the 20 mg/kg threshold, with these two methods (buckwheat flour: R5: 19.1 mg/kg−G12: 24.3 mg/kg; falafel mix: R5: 21.7 mg/kg–G12: 13.9 mg/kg).

## 4. Conclusions

In summary, it can be said that results obtained with the G12 antibody ELISA assay are comparable to the official R5 method. Real-life sample results by the official R5 methods could be confirmed with the G12 method and it is therefore a suitable method. The in-house protein extraction procedure could also be confirmed as satisfying, as no big deviations between extracts a and b could be observed.
